# Stimulants

**Published:** 1870-12

**Authors:** 


### STIMULANTS.

That man is a maniac, a deliberate suicide, who drinks tea, coffee or ardent spirits of any kind, to induce him to perform a work in hand, and when he feels too weak to go through with it without such aid. This is the reason that the majority of great orators and public favorites die drunkards. The pulpit, the bench, the bar, the forum, have contributed their legions of victims to drunken habits. The beautiful woman, the sweet singer, the conversationalist, the periodical writers, have filled but too often a drunkard's grave. Now that the press has become such a great power in the land, when the magazine must come out on a certain day and the daily newspapers at a fixed hour, nothing waits, everything must give way to the inexorable call for copy, and, sick or well, disposed or indisposed, asleep or awake, that copy must come; the writer must compose his article, whether he feels like it or not, and if he is not in the vein for writing, he must whip himself up to it by the stimulus of drink. Some of the greatest writers of the century have confessed to the practice, on urgent occasions, of taking a sip of brandy at the end of every written page, or even oftener—Lord Byron at the end of every paragraph sometimes!

It may have escaped the general reader's notice that more men have died young, who have been connected with the New York Press, within ten years, and that too from intemperance, than in all the other educational callings put together; young men whose talents have been of the very first order, and gave promise of a life of usefulness, honor and eminence. The best possible thing for a man to do, when he feels too tired to perform a task, or too weak to carry it through, is to go to bed and sleep a week, if he can; this is the only true recuperation of brain power; the only actual renewal of brain force; because during sleep the brain is in a sense at rest, in a condition to receive and appropriate particles of nutriment from the blood which take the place of those which have been consumed in previous labor, since the very act of thinking consumes, burns up solid particles, as every turn of the wheel or screw of the splendid steamer is the result of the consumption by fire of the fuel in the furnace. That supply of consumed brain substance can only be had from the nutrient particles in the blood which were obtained from the food eaten previously, and the brain is so constituted, that it can best receive and appropriate to itself those nutrient particles during the state of rest, quiet and stillness of sleep. Mere stimulants supply nothing in themselves—they only goad the brain, force it to a greater consumption of its substance until that substance has been so fully exhausted that there is not power enough left to receive a supply; just as men are sometimes so near death by thirst or starvation, that there is not strength enough left to swallow anything, and all is over. This incapacity of the brain for receiving recuperative particles sometimes comes on with the rapidity of a stroke of lightning, and the man becomes mad in an instant; in an instant falls in convulsions, in an instant loses all sense, and he is an idiot. It was under circumstances of this very sort, in the very middle of a sentence of great oratorical power, one of the most eminent minds of the age forgot his idea, pressed his hand against his forehead, and after a moment's silence said, "God, as with a sponge, has blotted out my mind." Be assured, reader, "There is rest for the weary" only in early and abundant sleep, and wise and happy are they who have firmness enough to resolve that "By God's help I will seek it in no other way."

But let it be understood that under all circumstances of extraordinary exhaustion from over-effort of mind or body, stimulants of tea, coffee or spirits are imperatively demanded at the earliest moment afterwards, to prevent the system from going down to that point of weariness when the body loses the power of rest, and the mind the power to sleep, for many have found themselves at times too tired to sleep. But then stimulants are taken, not to enable the person to attempt more work, but to enable him to take rest and sleep, by preventing the system from getting so low as to banish both.

